# Optimal cut-off value for equol-producing status in women: The Japan Nurses’ Health Study urinary isoflavone concentration survey

**DOI:** 10.1371/journal.pone.0201318

**Published:** 2018-07-26

**Authors:** Yuki Ideno, Kunihiko Hayashi, Junko Nakajima-Shimada, Yoko Onizuka, Mikiko Kishi, Tomomi Ueno, Shigeto Uchiyama

**Affiliations:** 1 Gunma University Initiative for Advanced Research, Maebashi City, Gunma, Japan; 2 Graduate School of Health Science, Gunma University, Maebashi City, Gunma, Japan; 3 Center for Medical Education, Gunma University, Maebashi City, Gunma, Japan; 4 Saga Nutraceuticals Research Institute, Otsuka Pharmaceutical Co., Ltd. Kanzaki-gun, Saga, Japan; International University of Health and Welfare, School of Medicine, JAPAN

## Abstract

Equol is one of the most active soy isoflavones. When the association between soy food intake in daily life and health outcomes is examined in epidemiological studies, it is important to define the equol-producing status of each individual. However, few studies have assessed equol-producing status without a soy challenge test. To determine a robust cutoff criterion for equol producer classification in observational studies, we conducted a urinary isoflavone concentration survey in daily life among women. Furthermore, we examined the association between eating habits regarding soy foods and equol-producing status. A total of 4,412 participants were included in the analyses. Urinary isoflavones were analyzed using a high-performance liquid chromatography method. We examined the distribution of the log_10_ equol/daidzein ratios, finding a mixture of two normal distributions, corresponding to equol producer and non-producer subpopulations. Applying a finite mixture model, we estimated the means, standard deviations, and mixing proportions of these two distributions. The estimation was carried out using the SAS NLIN procedure. The optimal cutoff point for the log_10_ equol/daidzein ratio in the study population was determined to be −1.42, according to the estimated parameters of the mixture distribution. Based on this criterion, 1,830 (41.5%) of the participants were identified as equol producers. Compared with non-consumers of soy foods, consumers of soy foods had significantly higher odds of being equol producers. Using log_10_-transformed equol/daidzein ratios ≥ −1.42 to define equol producers among Japanese women is reasonable and suitable for determining equol-producing status in epidemiological studies. We found that soy food eating habits were associated with equol-producing status. Further investigation is required to evaluate associations between equol-producing status in daily life and health outcomes. The results of this study suggest the best cutoff point to use in the definition of equol-producing status in daily life.

## Introduction

Soy food consumption has been linked to decreased risks of chronic diseases such as cancer, heart disease, and osteoporosis among women [1–3]. Soybeans are a rich source of isoflavones, including daidzein, genistein, glycitein, and equol. To different extents, these biologically active compounds may play a role in the prevention of the abovementioned diseases. Recently, interest has increased in equol, one of the most active soy isoflavones.

Equol is a metabolite formed in the biotransformation of the soy isoflavone daidzein by intestinal bacteria. Equol has a similar chemical structure to oestradiol [4], and equol has a higher affinity for binding to the estrogen receptor-β than does its precursor, daidzein [5]. It is expected that equol has higher estrogen activity, and the effects of soy foods on health outcomes such as decreasing vasomotor symptoms [6] and bone resorption [7], might differ depending on equol-producing status. Not everyone is able to produce equol. For example, only 20% to 35% of the Western adult population has been reported to be capable of producing equol when fed soy foods or isoflavone supplements [8–11]. Equol producers have been found to make up 50% to 60% of the population of adults living in Asian countries where soy is consumed regularly [12,13]. When the association between soy food intake in daily life and health outcomes is examined in epidemiological studies, it is important to define the equol-producing status of each individual.

Although equol-producing status is determined from urinary or serum equol concentration, there are no established cutoffs for defining an equol producer. Some studies have used the detection limit for equol as a cutoff [14–16], and others have used arbitrary cutoffs [8,9,17]. These dissimilarities may result from differences in the analytical methods used to measure equol. Furthermore, using an absolute equol concentration threshold is problematic because the concentration of equol in serum or urine is influenced by the pharmacokinetic behavior and bioavailability of isoflavones [18]. To solve these problems, Setchell and Cole suggested defining equol producers using the cutoff of log_10_-transformed urinary ratio ≥ −1.75 [19]. Franke et al. found that Setchell and Cole’s cutoff was useful, even when using overnight urine samples instead of 24-h urine samples, which are difficult to obtain in large-scale studies [20].

Few studies have assessed equol-producer status using urinary isoflavone concentration in a daily life setting without a soy challenge test, during which participants are given soy products such as soy milk or soy bars for a few consecutive days before urine samples are collected. To determine a robust equol cutoff criterion for equol producer classification in observational epidemiological studies, we conducted a urinary isoflavone concentration survey in daily life among women participating in the Japan Nurses’ Health Study (JNHS). Furthermore, we examined the association between eating habits regarding soy foods and equol-producing status.

## Materials and methods

### Study population

This study surveyed 4,472 women aged 33−86 years, who participated in the Urinary Isoflavone Concentration Survey of the JNHS. Participants were excluded from the analyses for the following reasons: daidzein concentration undetermined in peak identification (n = 24), equol concentration undetermined in peak identification (n = 17), daidzein not detected (n = 1), or equol supplement use (n = 21). A total of 4,412 participants were included in the statistical analyses. The mean ± standard deviation for age was 54.9 ± 8.5 years. Of the women in our study population, 32.5% were pre-menopausal, 54.5% were post-menopausal, and 13.0% had unclear menopausal status.

The study was carried out May–December 2015. Participants were given urine collection kits and asked to provide early morning urine samples. In addition, participants were asked to complete a self-administered questionnaire (S1 Fig) that collected information on lifestyle factors such as diet (in the last year and the last 48 hours) and supplement use in the last week. The short-form food frequency questionnaire used in this study has been validated [21]. Participants mailed these questionnaires and samples to our laboratory at Gunma University, and the urine aliquots were stored at −70°C until the isoflavone analysis.

### Urinary isoflavone analysis

Daidzein and equol levels in urine were measured by Saga Nutraceuticals Research Institute, Otsuka Pharmaceutical Co., Ltd., Japan, using high-performance liquid chromatography (HPLC; Nexera X2; Shimadzu, Japan) with a type C18 column (CAPCELL CORE C_18_, 2.7 μm 4.6 Φ × 100 mm; Shiseido Co., Ltd., Japan) and an SPD-M30A PDA and RF-20Axs detection system (Shimadzu, Japan). Beta-glucuronidase/sulfate was added to a 0.1 mL urine sample. The samples with beta-glucuronidase/sulfate were incubated for 30 minutes at 37 degrees. The aglycone fractions of the isoflavones and their metabolites were extracted using OASIS HLB microelution plates (Waters, USA) prior to the HPLC analysis. Quantitation was performed by means of UV response (254 nm and 280 nm) for daidzein and fluorescence response (Em: 255 nm, Ex: 310 nm) for equol. In each batch of samples, laboratory precision was assessed by analyzing the standard solution. The limits of detection (LODs) were 0.026 nmol/mL for daidzein and 0.008 nmol/mL for equol.

### Determination of equol-producing status

**Fig 1:**
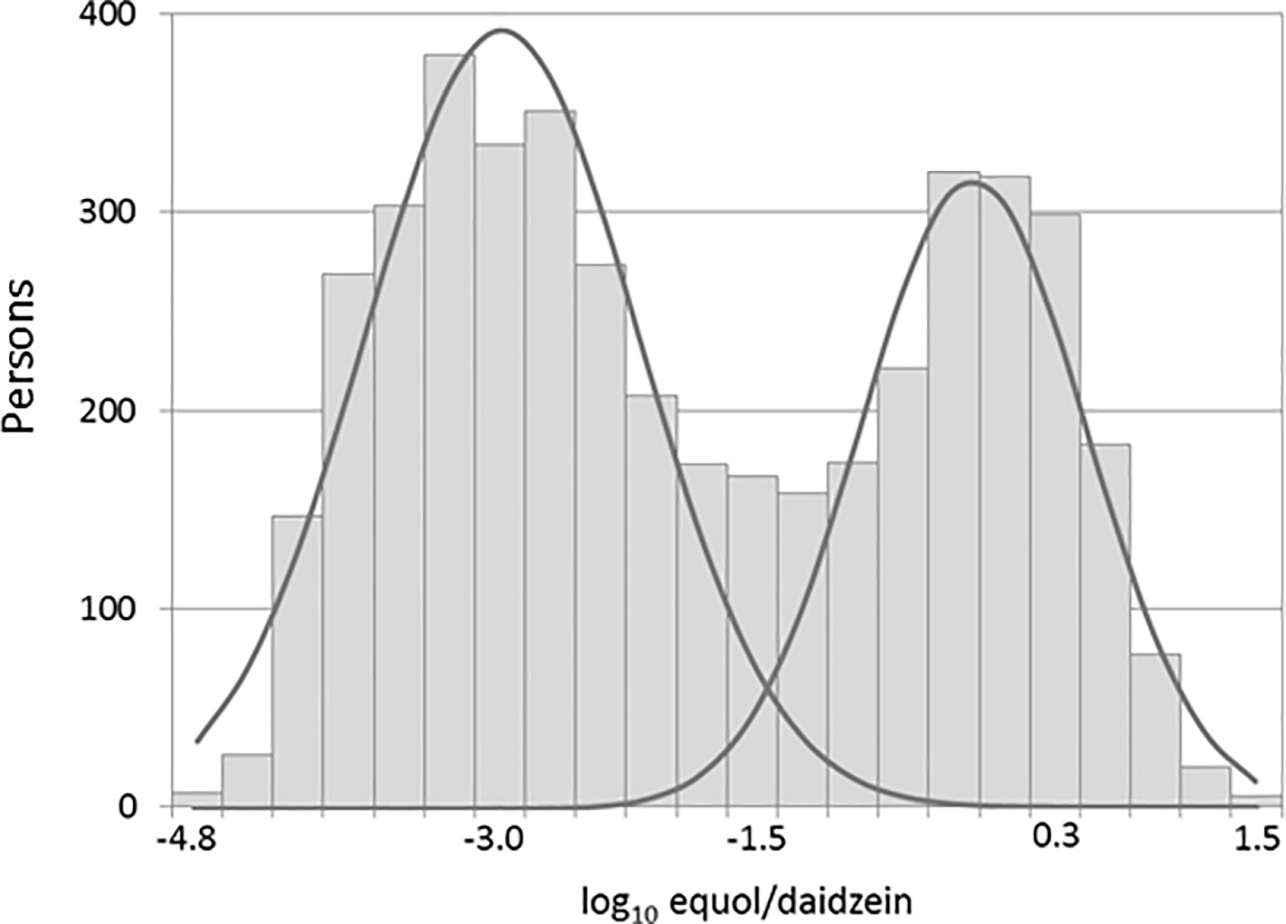
Distribution of log_10_ equol/daidzein ratio. The urinary isoflavone data were expressed using the log_10_ equol/daidzein ratio. The distribution of this ratio showed a mixture of two normal distributions, considering the left-hand distribution to represent non-producers and the right-hand distribution to represent equol producers.

We applied a finite mixture model—specifically, a two-class univariate normal mixture model—to estimate the cutoff point for defining equol-producing status. Du et al. have suggested that this model may be used to evaluate the threshold in the absence of other diagnostic tests as gold standards [22]. Log_10_-transformed product/precursor ratios (log_10_ equol/daidzein) were used as Setchell and Cole have described previously [19]. First, participants with equol not detected were assigned the equol value of LOD/2 (0.004 nmol/mL). Next, the urinary isoflavone data were expressed using the log_10_ equol/daidzein ratio. The distribution of this ratio showed a mixture of two normal distributions, corresponding to equol producer and non-producer subpopulations (Fig 1). This was modelled as a mixture of two normal distributions.

The probability density function for this model is

$$f(x)=pg_{1}(x)+(1-p)g_{2}(x),$$ (1)

where *f*(*x*) is the aggregate density function, *g*_1_(*x*) and *g*_2_(*x*) are the component densities of the two classes, and *p* and (1−*p* are the mixing proportions that designate the fraction of participants originating from each component. The component densities are defined to be normally distributed so that

$$g_{1}(x)={\left(2\pi \sigma_{1}^{2}\right)}^{-1/2}e^{-\left[{\left(x_{i}-\mu_{1}\right)}^{2/{2\sigma }_{1}^{2}}\right]}$$ (2)

$$g_{2}(x)={\left(2\pi \sigma_{2}^{2}\right)}^{-1/2}e^{-[{\left(x_{i}-\mu_{2}\right)}^{2/{2\sigma }_{2}^{2}}]}.$$ (3)

Estimation of unknown parameters was carried out using the SAS NLIN procedure. It was assumed that the two normal distributions in the mixture represented either equol non-producers or equol producers. The optimal cutoff log_10_ equol/daidzein value was defined as the point where the probability of misclassification (false positive and false negative probabilities weighted by the mixing proportions), based on the estimated mixture distribution, reached its minimum value.

### Data analysis

The prevalence of equol producers was calculated using the criterion described above. The Cochran–Armitage test was adopted to evaluate the association between age group (< 50, 50–59, and ≥ 60 years of age) and equol-producing status, and a chi-squared test was used to evaluate the association between menopausal status and equol-producing status.

The effects of soy food eating habits (tofu, *natto* (fermented soybeans), miso soup, and soy milk) on equol-producing status were examined using logistic regression analyses. Responses to questions about the frequency of the consumption of soy foods were categorized as “≤ 1 day/week,” “2–3 days/week,” or “≥ 4 days/week.” Participants who responded “≤ 1 day/week” to questions on all four soy foods were classified as non-consumers of soy foods. We first estimated age-adjusted odds ratios (OR) and 95% confidence intervals (CI) for consumers of soy foods. We then estimated age-adjusted ORs and 95% CIs for the consumption frequency categories among consumers of soy foods. A value of p < 0.05 was considered to be statistically significant. All analyses were performed using SAS version 9.4 statistical software (SAS Institute, Cary, NC, USA).

The protocol for this study was reviewed and approved by the Ethics Review Board for epidemiological studies of the Faculty of Medicine, Gunma University. Written informed consent was obtained from all participants.

## Results

### Determination of equol-producing status

To determine equol-producing status, the urinary isoflavone data were expressed using the log_10_ equol/daidzein ratio. The distribution of this ratio is shown in Fig 1, where two distributions are clearly displayed. We considered the left-hand distribution to represent non-producers and the right-hand distribution to represent equol producers. Applying a finite mixture model, we estimated the means (m1, m2), standard deviations (s1, s2), proportions (p, 1 –p) of these two distributions. Estimates and approximate 95% confidence intervals (CIs) were as follows (Fig 1):

m1=−2.990(approximate95%CI:−2.993–−2.986)

s1=0.815(0.811–0.818)

m2=−0.177(−0.181–−0.174)

s2=0.671(0.666–0.675)

p=0.587(0.586–0.587)

The optimal cutoff point for the log_10_ equol/daidzein ratio was determined to be −1.42, using the minimum value of the probability of misclassification as the criteria for optimization (false positive probability = 0.03, false negative probability = 0.03, and probability of misclassification = 0.03 × 0.587 + 0.03 × 0.413 = 0.018 + 0.012 = 0.030). We defined participants with log_10_ equol/daidzein ratios ≥ −1.42 as equol producers and participants with log_10_ equol/daidzein ratios < −1.42 as equol non-producers. Table 1 shows the cutoff points for populations that have different proportions of subgroups.

**Table 1:** Estimations of cutoff points for populations that have different proportions.

Population	Proportion of equol producers	Proportion of equol non producers	cutoff point	False positive probabiloty	False negative probabiloty	Probability of misclassification
JNHS^a^	0.413	0.587	-1.42	0.0270	0.0323	0.0300
50%^b^	0.500	0.500	-1.49	0.0324	0.0256	0.0290
20%^c^	0.200	0.800	-1.24	0.0159	0.0566	0.0240

^a^JNHS is the population of this study.

^b^50% is the population assumed that has 50% equol producers and 50% non-producers.

^c^20% is the population assumed that has 20% equol producers and 80% non-producers.

### Equol producers in the JNHS cohort

Based on the criterion described above, we identified 1,830 (41.5%) equol producers. The prevalence of equol producers in each of the age groups was as follows: 38.6% among those aged < 50 years, 41.4% among those aged 50–59 years, 44.3% among those aged ≥ 60 years (Table 2). There was a trend toward a higher prevalence with older age (p < 0.01, Cochran–Armitage test). The prevalence of equol producers among pre-menopausal women and post-menopausal women was 40.3% and 43.0%, respectively (Table 2). There were no significant differences between pre-menopausal women and post-menopausal women (p = 0.10, chi-squared test).

**Table 2:** Characteristics of the study participants.

		equol producers^a^		equol non-producers^a^		P-value
		n = 1,830		n = 2,582		
age group	< 50 years	502	(38.6)	799	(61.4)	< 0.01^b^
	50–59 years	715	(41.4)	1,013	(58.6)	
	≥ 60 years	613	(44.3)	770	(55.7)	
menopausal status	pre-menopause	579	(40.3)	857	(59.7)	0.10 ^c^
	post-menopause	1,034	(43.0)	1,371	(57.0)	
	unclear	217		354		
soy food eating habits	non-consumers of soy foods	165	(36.2)	291	(63.8)	0.01 ^c^
	consumers of soy foods	1,577	(42.4)	2,140	(57.6)	
	missing	88		151		
	**tofu**					
	non-consumers of soy foods	165	(36.2)	291	(63.8)	< 0.01^b^
	≤ 1 day/week^d^	340	(39.9)	512	(60.1)	
	2–3 days/week	690	(40.9)	997	(59.1)	
	≥ 4 days/week	547	(46.4)	631	(53.6)	
	***natto***					
	non-consumers of soy foods	165	(36.2)	291	(63.8)	< 0.01^b^
	≤ 1 day/week^d^	804	(40.9)	1,160	(59.1)	
	2–3 days/week	416	(43.0)	552	(57.0)	
	≥ 4 days/week	357	(45.5)	428	(54.5)	
	**miso soup**					
	non-consumers of soy foods	165	(36.2)	291	(63.8)	< 0.01^b^
	≤ 1 day/week^d^	287	(40.0)	430	(60.0)	
	2–3 days/week	427	(40.1)	638	(59.9)	
	≥ 4 days/week	863	(44.6)	1,072	(55.4)	
	**soy milk**					
	non-consumers of soy foods	165	(36.2)	291	(63.8)	0.17 ^b^
	≤ 1 day/week^d^	1,160	(42.4)	1,577	(57.6)	
	2–3 days/week	162	(42.9)	216	(57.1)	
	≥ 4 days/week	255	(42.4)	347	(57.6)	

^a^Values are the numbers of participants, with percentages in parentheses.

^b^Cochran–Armitage test

^c^Chi-squared test

^d^Excluding participants who reported “≤ 1 day/week” for all four soy foods

To examine the associations between soy food eating habits and equol-producing status, age-adjusted logistic regression analyses were performed. Compared with non-consumers of soy foods, consumers of soy foods had a significantly higher odds of being equol producers (age-adjusted OR = 1.27, 95% CI 1.04–1.56).

## Discussion

The most beneficial aspect of using the log_10_-transformed equol/daidzein ratio is the simplicity of detecting equol producers without a soy challenge test in daily life. We can easily determine equol-producing status as long as urinary daidzein is detected. Moreover, if urinary daidzein is not detected, there is little exposure to equol in daily life, regardless of the individual’s equol-producing status.

Compared with the cutoff point of log_10_-transformed equol/daidzein ratio of −1.75 used by Setchell and Cole (n = 41) [19], we estimated a log_10_-transformed equol/daidzein ratio of −1.42 as the optimal cutoff for defining equol-producing status (n = 4,412) in our study population. There are several reasons that may explain why the result from the present study differs from that of the previous study. First, our sample size was large enough to find the cutoff statistically. In addition, because the prevalence of equol producers in the Japanese population was about 50%, the size of the two distributions considered to define equol producers and non-producers was almost the same. In contrast, when it was assumed that the prevalence of equol producers was 20%, as is the case in Western adult populations, the optimal cutoff point was −1.24 (Table 1). Therefore, the cutoff of −1.42 seems to be more objective and accurate for Japanese women than the cutoff used by Setchell and Cole. Second, our survey was conducted without a soy challenge. For observational epidemiological studies, equol-producing status in daily life is more important than equol-producing ability detected with a soy challenge test. It seems that our suggested criterion is more reasonable and suitable for epidemiological studies.

Third, compared with 24-h urine samples in the study of Setchell and Cole [19], we used early morning urine samples. Although 24-h urine samples are superior to spot urine samples, 24-h samples are difficult to obtain in large-scale studies. Franke et al. ascertained that defining equol producers using the equol to daidzein ratio was useful in large-scale studies, even when using overnight urine samples instead of 24-h urine samples [20]. Additionally, in our study, the LOD for equol was set at 0.008 nmol/mL, compared with 0.04 nmol/mL in the previously used HPLC method, to improve the accuracy of detecting equol levels [14].

The prevalence of equol producers in the JNHS cohort was 41.5%. Few studies have assessed equol-producing status using urine samples in a daily life setting without a soy challenge. To our knowledge, only three previous studies have shown the equol-producing prevalence for Japanese women in daily life: Nagata et al. [14] identified 20% of their participants (n = 419) as equol producers, Liu et al. [16] identified 39% of their participants (n = 500) as equol producers, and Yoshikata et al. [17] identified 32% of their participants (n = 743) as equol producers. Compared with these studies, the prevalence found in the JNHS cohort was slightly higher. Yoshikata et al. [17] defined equol producers as having urinary equol levels higher than 1.0 μM, and Nagata et al. [14] and Liu et al. [16] used the detection limits for equol as a cutoff (0.04 nmol/mL and 20 ng/mL, respectively). However, those definitions are not suitable for an epidemiological study setting without a soy challenge test. In our view, it is important to consider the definition of equol producers used when comparing the prevalence of equol producers across populations or studies. This highlights the need for a standard definition using the proper log_10_ equol/daidzein ratio cutoff. The main purpose of our study was to offer a gold standard for equol-producing status in daily life.

We found that soy food eating habits were associated with equol-producing status. Even if people have the ability to produce equol, they cannot produce it without its precursor, daidzein. Therefore, it is speculated that people who consume soy foods are more likely to develop or maintain the ability to produce equol than are non-consumers of soy foods. The finding of an association between soy food eating habits and equol-producing status supports previous reports that the prevalence of equol producers is higher in Asian populations than in Western populations because of the soy food eating habits in Asian countries. Ko TF et al. [23] highlighted that copresence of the active intestinal microorganisms and dietary daidzein is essential for urinary equol excretion. They suggested that the continuous consumption of soy foods may promote some specific equol-converting microbial inhabitants to adapt and grow gradually, and induces the non-producer into producer. However, the participants in this study entered this survey voluntarily, and it is possible that most of them were more interested in their own health and consumed more soy foods, compared with the general population.

The strength of this study is the very large number of participants. As a result, we were able to calculate a log_10_-transformed equol/daidzein ratio of −1.42 as the cutoff for defining equol-producing status statistically. However, there are some limitations. First, this study was limited in that the observations were restricted to Japanese women, whose soy consumption is relatively high, which precludes generalization to the Western population. However, our sample size was very large and included a variety of participants, ranging from women with a very high level of soy food consumption to those who hardly consume soy foods. Therefore, we believe that our criterion assuming the prevalence of equol producers in Western populations is also applicable to Western women. Second, we investigated the frequency of consumption of only four soy foods, namely tofu, *natto*, miso soup, and soy milk. These are certainly notable examples of Japanese soy foods, but Japanese women also eat other soy foods, such as green soybeans, soybean flour, and deep-fried tofu. Although consumers of soy foods had a significantly higher probability of being equol producers, the prevalence of equol producers among non-consumers of soy foods and consumers of soy foods was 36.2% and 42.4%, respectively, which does not indicate a very large difference. Although reliability and validity of the soy food frequency questionnaire has been previously examined [21], it is possible that participants who consumed soy foods other than tofu, *natto*, miso soup, and soy milk were classified as non-consumers of soy foods. Investigating eating habits in terms of other soy foods and the influence of these soy foods on equol-producing status are problems for future analysis.

In summary, we conducted a urinary isoflavones concentration survey in daily life among Japanese women and defined participants with log_10_-transformed equol/daidzein ratios ≥ −1.42 as equol producers, and participants with log_10_ equol/daidzein ratios < −1.42 or with equol not detected as equol non-producers. Based on this criterion, we found that soy food eating habits were associated with equol-producing status. Further investigation is required to evaluate associations between equol-producing status in daily life and health outcomes. The results of this study suggest the best cutoff point to use in the definition of equol-producing status in daily life.

## Supporting information

S1 Fig Questionnaire (in Japanese). (PDF)
