# Supplementary material for: Optimal cut-off value for equol-producing status in women: The Japan Nurses’ Health Study urinary isoflavone concentration survey
Source: PLoS One. 2018 Jul 26;13(7):e0201318. doi: 10.1371/journal.pone.0201318 (PMC6062095; doi:10.1371/journal.pone.0201318)
Supplement: S1 Fig — (PDF) [file pone.0201318.s001.pdf]

ID：

JNHS 尿中イソフラボン測定調査　調査票

この調査票に記入した後に、採尿をしてください。

下記の空欄の部分に必要事項を記入し、該当する□にチェック☑してください。

記入日：2015 年　　月　　日

問１．現在の身長と体重を記入してください。

現在の身長	cm	現在の体重	kg

問２．採尿日前の一週間の薬剤使用についてお答えください。

採尿日前の一週間に、処方薬や市販薬を使用しましたか		□ はい　□ いいえ ⇒ 問３へ	
抗生物質		□使用あり	□使用なし
女性ホルモンを含む薬剤	低用量ピル・経口避妊薬	□使用あり	□使用なし
	ホルモン補充療法	□使用あり	□使用なし
その他の処方薬・市販薬	（使用した薬剤名）		
	（使用した薬剤名）		
□使用あり　□なし	（使用した薬剤名）		

問３．採尿日前の一週間のサプリメントや健康食品についてお答えください。

	商品名	メーカー	最後に摂取した日
サプリメントや健康食品（大豆製の菓子［SOYJOY 等］を含む）を、過去一週間に、摂取しましたか			2015 年　月　日
			2015 年　月　日
			2015 年　月　日
			2015 年　月　日
□摂取あり　□なし			2015 年　月　日

参考例：商品名（メーカー）
大豆イソフラボン（ファンケル、サントリー、ネイチャーメイド、DHC 等）、エクエル、エクオールサプリ（大塚製薬、株式会社アドバンストメディカルケア）、レッドクローバー（ネイチャーズウェイ、NOW、わかさ生活等）、葛根湯（メーカー問わず）、大豆プロテイン（メーカー問わず）、プラセンタ（メーカー問わず）など

問 4. 月経状態、妊娠などについて、お答え下さい。

妊娠状況	□現在、妊娠中である　□いいえ
出産経験	□ なし　□ あり（出産回数:　　回 ）
授乳経験	□授乳経験あり（□現在、授乳中である）　□授乳経験なし
月経状態	閉経前 □ 定期的（　　日間隔）　□ 不定期<br>※前回の月経日　:　　年　月　日ごろから　月　日<br>※前々回の月経日:　　年　月　日ごろから　月　日<br>閉経後 □ 最終日:　　年　　月頃　または　何年前　　年前

問 5. 採尿前 48 時間、および過去 1 年間の食生活についてお答えください。

注意：採尿のために日頃の食事内容を変える必要はありません。

	採尿前 48 時間の摂取状況	過去 1 年間のおおよその食物摂取頻度 もっともあてはまる番号に○					
	摂取ありは、□にチェック	全くなし・ほとんどなし	週 1 回程度	週 2〜3 日	週 4〜5 日	ほぼ毎日	毎日 2 回以上
牛肉	□	1	2	3	4	5	6
豚肉	□	1	2	3	4	5	6
鶏肉	□	1	2	3	4	5	6
魚	□	1	2	3	4	5	6
緑黄色野菜	□	1	2	3	4	5	6
果物	□	1	2	3	4	5	6
牛乳・乳製品	□	1	2	3	4	5	6
豆腐	□	1	2	3	4	5	6
納豆	□	1	2	3	4	5	6
味噌汁	□	1	2	3	4	5	6
豆乳・豆乳製品	□	1	2	3	4	5	6
アブラナ科野菜＊	□	1	2	3	4	5	6

＊アブラナ科野菜：大根、こまつな、ブロッコリー、はくさい、など

問 6. 体調の確認：この 2 日間、下痢の症状がありますか。

下痢	□ なし　（下痢症状がある場合には、症状が回復してから採尿して下さい）

調査票の記入、有難うございました。それでは、採尿をよろしくお願いします。

採尿日時：　月　日（午前・午後　時）

注意：採尿は、基本的に、睡眠後、起床直後に行ってください。
